# HIV-1 elite controllers present a high frequency of activated regulatory T and Th17 cells

**DOI:** 10.1371/journal.pone.0228745

**Published:** 2020-02-05

**Authors:** Diogo G. Caetano, Hury H. S. de Paula, Gonzalo Bello, Brenda Hoagland, Larissa M. Villela, Beatriz Grinsztejn, Valdilea G. Veloso, Mariza G. Morgado, Monick L. Guimarães, Fernanda H. Côrtes

**Affiliations:** 1 Laboratório de AIDS e Imunologia Molecular, Instituto Oswaldo Cruz–IOC, FIOCRUZ, Rio de Janeiro, Brazil; 2 Instituto Nacional de Infectologia Evandro Chagas—INI, FIOCRUZ, Rio de Janeiro, Brazil; University Hospital Zurich, SWITZERLAND

## Abstract

HIV-1 infection is characterized by generalized deregulation of the immune system, resulting in increased chronic immune activation. However, some individuals called HIV controllers (HICs) present spontaneous control of viral replication and have a more preserved immune system. Among HICs, discordant results have been observed regarding immune activation and the frequency of different T cell subsets, including Treg and Th17 cells. We evaluated T cell immune activation, differentiation and regulatory profiles in two groups of HICs—elite controllers (ECs) and viremic controllers (VCs)—and compared them to those of cART-treated individuals (cART) and HIV-1-negative (HIV-neg) individuals. ECs demonstrated similar levels of activated CD4^+^ and CD8^+^ T cells in comparison to HIV-neg, while cART and VCs showed elevated T cell activation. CD4^+^ T cell subset analyses showed differences only for transitional memory T cell frequency between the EC and HIV-neg groups. However, VC individuals showed higher frequencies of terminally differentiated, naïve, and stem cell memory T cells and lower frequencies of transitional memory and central memory T cells compared to the HIV-neg group. Among CD8^+^ T cell subsets, ECs presented higher frequencies of stem cell memory T cells, while VCs presented higher frequencies of terminally differentiated T cells compared to the HIV-neg group. HICs showed lower frequencies of total Treg cells compared to the HIV-neg and cART groups. ECs also presented higher frequencies of activated and a lower frequency of resting Treg cells than the HIV-neg and cART groups. Furthermore, we observed a high frequency of Th17 cells in ECs and high Th17/Treg ratios in both HIC groups. Our data showed that ECs had low levels of activated T cells and a high frequency of activated Treg and Th17 cells, which could restrict chronic immune activation and be indicative of a preserved mucosal response in these individuals.

## Introduction

HIV-1 controllers (HICs) are a rare group of HIV-1-infected individuals able to spontaneously control viral replication in the absence of combined antiretroviral therapy (cART). Classically, these individuals are divided into two groups: Elite controllers (ECs), who are able to keep plasma viral loads below the detection limit of clinical assays (currently < 40 HIV-1 RNA copies/ml), and viremic controllers (VCs), who present plasma viral loads < 2,000 HIV-1 RNA copies/ml [1].

HIV-1 infection is characterized by generalized deregulation of the immune system, resulting in high levels of chronic immune activation [2,3], which has been described as a state of increased cellular turnover, cell cycle deregulation and establishment of an inflammatory setting [2,4] that is not fully normalized even after initiation of cART [5–8]. Moreover, alterations in the frequency of different T cell subsets, leading to an increase in effector or fully differentiated T cells [2,4,9–11] and a decrease in naïve T cells [2,10,12,13], have also been observed as a consequence of the chronic immune activation. Despite the viremia control, some HICs present higher levels of immune activation and inflammation than HIV-1-uninfected individuals [14–16], mainly the VC individuals [17,18].

In addition to alterations in the frequency of naïve, effector and memory T cells, the chronic phase of HIV infection has been associated with an increased frequency of regulatory T cells (Treg) [19–28], which are a subset of CD4^+^ T cells that regulate the immune response and the proliferation of effector T cells [29–31]. In the context of HIV-1 infection, the immunosuppressive function of Treg cells has been described to have both detrimental and protective effects on disease progression. Higher frequencies of Treg cells correlate with high plasma viral load and progression to AIDS [19–28], while lower frequencies have been observed for HICs/long-term nonprogressors (LTNPs) [32–35] and cART-treated patients [25,26,28,35,36] and are associated with an increase in viral-specific CD8^+^ T cell response [37–41]. On the other hand, higher frequencies of Treg cells are associated with a decrease in the systemic immune activation [28,35,42].

Another T cell subset affected during HIV-1 infection is Th17 cells. These cells are enriched in the mucosal tissues and classically produce a set of proinflammatory cytokines (e.g., IL-17, IL-22, IL-21) [43–45] that enhance the expression of antimicrobial peptides [46], recruit neutrophils [47,48] and induce epithelial regeneration [49], thus playing an essential role in the host defense against microbial pathogens and maintenance of epithelial integrity at mucosal sites. Th17 cells are preferentially depleted during the acute phase in pathogenic SIV models [50–52] but preserved in nonpathogenic infection [51,53], and a lower frequency of these cells is observed during the chronic phase in HIV-infected patients with progressive disease [53–56].

Despite their opposite functions, both the Treg and Th17 subsets are derived from a common progenitor cell, with their formation determined by the expression levels of IL-6 and TGF-β [57]. Thus, inverse and reciprocal alterations in both subsets have been observed in the context of HIV-1 infection, and the loss of the balance between these two populations has been associated with disease progression [32–34,51]. In contrast, higher Th17/Treg ratios have been observed in ECs compared to typical progressors [32–34].

In the present study, we aimed to evaluate parameters related to the immune activation, memory T cells, and regulatory T cells in HICs and the distribution of different T cell subsets involved in the immune response. Beyond the frequencies of activated T cells, we evaluated the frequencies of naïve, stem cell memory, central memory, transitional memory, effector memory and terminally differentiated T cells in both ECs and VCs, comparing with the frequencies observed for HIV-negative individuals and cART-treated individuals. We also evaluated the frequencies of total Tregs and their different subsets, as well as the frequencies of Th17 cells to assess the Th17/Treg balance. Our data showed that ECs had low levels of activated T cells and a high frequency of activated Treg cells, which could contribute to lower immune activation in these individuals. Additionally, a higher frequency of Th17 cells in ECs might be indicative of preserved mucosal response resulting in low microbial translocation and immune activation.

## Materials and methods

### Study population and ethical statement

Twenty-seven HICs were selected from the Instituto Nacional de Infectologia Evandro Chagas/Fiocruz (INI-Fiocruz) HIV-1 cohort for this study and were classified into two groups: (1) ECs (n = 14) if the plasma viral load (VL) measurements were below the lower detection limit (<LDL) depending on the commercial method available during the clinical and laboratory follow-up (< 50–80 copies/ml) and (2) VCs (n = 13), if most (≥ 70%) VL measurements were >LDL and <2,000 copies/ml. Occasional VL measurements above the upper limits were accepted during the follow-up of the EC and VC groups. A group of HIV-1-infected individuals on cART with a suppressed VL for at least two years (cART; n = 18) and a group of HIV-1-uninfected individuals (HIV-neg; n = 18) were also included as controls. All participants provided written informed consent, and both the INI-Fiocruz Ethical Committee Board and the Brazilian National Human Research Ethics Committee (CONEP 840/2008) approved the study.

### Sample preparation

Peripheral blood mononuclear cells (PBMCs) were isolated from whole blood by Histopaque-1077 (Sigma-Aldrich, USA) density gradient centrifugation and stored in liquid nitrogen until use.

### CD4^+^ and CD8^+^ T cell count and plasma VL determination

Absolute CD4^+^ and CD8^+^ T cell counts were obtained from whole blood using the MultiTest TruCount-kit and the MultiSet software on a FACSCalibur flow cytometer (BD Biosciences, USA). Plasma HIV-1 viral loads of the samples corresponding to the time points analyzed in the present study were measured using the Abbott RealTime HIV-1 assay (Abbott Laboratories, Germany), with LDL of 40 copies/ml.

### Flow cytometry

For each patient, vials of 1x10^7^ cryopreserved PBMCs with viability >85% were thawed and rested overnight in RPMI 1640 (Sigma-Aldrich) supplemented with 10% fetal bovine serum (FBS, Gibco—Thermo Fisher Scientific, USA) at 37°C with 5% of CO_2_ and controlled humidity. For naïve, memory, effector and activated CD4^+^ and CD8^+^ T cell subsets, detailed below, PBMCs were stained with FVS450 (BD Biosciences, USA) for dead cells exclusion, and with anti-CD3 APC-H7, anti-CD4 PE-CF594, anti-CD8 APC, anti-CD45RA PE-Cy7, anti-CD27 BV510, anti-CCR7 Alexa Fluor 700, anti-CD95 PerCP-Cy5.5, anti- HLA-DR PE and anti-CD38 BB515 (all from BD Biosciences, USA). The T cell activation status was evaluated based on the analysis of CD38 and HLA-DR coexpression, while T cell subsets were classified as follows: naïve (TN: CD45RA^+^CCR7^+^CD27^+^CD95^-^), stem cell memory (TSCM: CD45RA^+^CCR7^+^CD27^+^CD95^+^), central memory (TCM: CD45RA^-^CCR7^+^CD27^+^), transitional memory (TTM: CD45RA^-^CCR7^-^CD27^+^), effector memory (TEM: CD45RA^-^CCR7^-^CD27^-^), and effector or terminally differentiated (TEFF: CD45RA^+^CCR7^-^CD27^-^). FMO controls were used to properly identify the CD45RA+, CCR7+,CD27+, CD38+ and HLA-DR+ populations.

For Treg and Th17 cell frequencies determination, PBMCs were stimulated with PMA and ionomycin (50 ng/ml and 1 μg/ml, respectively; Sigma-Aldrich, USA) in the presence of Golgi Stop (Human Th17/Treg Phenotyping Kit; BD Biosciences, USA) according to the manufacturer’s instructions, for five hours. The cells were stained with FVS450, anti-CD25-BB515, and anti-CD8-BV510 (all from BD Biosciences, USA). After, the cells were washed with staining buffer (2% of FBS in PBS) and fixed using the Human FoxP3 Buffer A (Human Th17/Treg Phenotyping Kit; BD Biosciences, USA). Subsequently, the cells were washed and incubated with a staining buffer at 4°C overnight. Then, the cells were permeabilized using Human FoxP3 Buffer C (Human Th17/Treg Phenotyping Kit; BD Biosciences, USA) and stained with anti-CD3-APC-H7, anti-CD45RA-PeCy7 and Human FoxP3 cocktail (Human Th17/Treg Phenotyping Kit; BD Biosciences, USA). Samples were acquired on the same day using a BD FACSAria^™^ IIu flow cytometer (BD Biosciences, USA), and analyses were performed with FlowJo software v.10.0.7 (Tree Star, USA). Th17 cells were defined as CD4^+^IL17^+^ T cells, while Treg cells were defined as CD4^+^CD25^high^Foxp3^+^ T cells, with the Treg subsets classified as follows: activated Treg (CD45RA^-^Foxp3^high^), non-suppressive Treg (CD45RA^-^Foxp3^low^) and resting Treg (CD45RA^+^Foxp3^low^). FMO controls were used to properly identify the CD45RA^+^, CD25^+^, IL17^+^, Foxp3^+^ populations.

### Statistics

Mann-Whitney tests were used to compare the frequencies of the above-cited T cell subsets among the studied groups. Correlations were calculated using Spearman regression. P-values < 0.05 were considered significant. All analyses were carried out using GraphPad Prism v.7.

## Results

### Clinical and demographic characteristics

The clinical and demographic characteristics of the studied groups are shown in Table 1. No significant difference in age was found between the groups, but ECs had a significantly higher frequency of women than VCs and cART (79% vs. 31% vs. 39%). The plasma VL had a median of 450 copies/ml in VCs, but undetectable levels were found in all ECs and cART. Higher CD4^+^ T cell counts were observed in ECs compared with cART (p = 0.0079). Detailed CD4+ T cells/mm3 and VL profiles of the ECs and VCs during the long-term follow-up were previously described [58,59]. ECs and VCs had medians of 8.5 and 10.4 years of HIV diagnosis time, respectively.

**Table 1:** Demographic and clinical characteristics of study participants.

	HIV-neg (n = 18)	cART (n = 18)	EC (n = 14)	VC (n = 13)
**Age, median** **[IQR]**	37.1 [29.80–49.55]	44.5 [38.28–50.05]	42.8 [37.60–58.8]	42.7 [37.60–47.05]
**Gender (%M)**	50	61	21	69
**Viral load (copies/ml), median [IQR]**	N/A	<40 [<40]	<40 [<40–87]	450 [224.0–881.5]
**CD4+ T cells count (cells/mm^3^), median [IQR]**	831 [741.80–1227]	853^a^ [745–1006]	1165 [888–1486]	830 [605–1365]
**Years since HIV-1 diagnosis, median** **[IQR]**	N/A	10.9 [8.7–15.1]	8.5 [4.0–15.4]	10.4 [5.4–14.55]

HIV-neg: HIV-1-uninfected individuals; EC: Elite controllers; VC: viremic controllers; cART: Chronic HIV-1 infected individual under cART and at least two years of VL below limit. N/A: not applicable. P-value was obtained using the Mann-Whitney test.

^a^ p = 0.0079, comparing EC with cART group.

**Fig 1:**
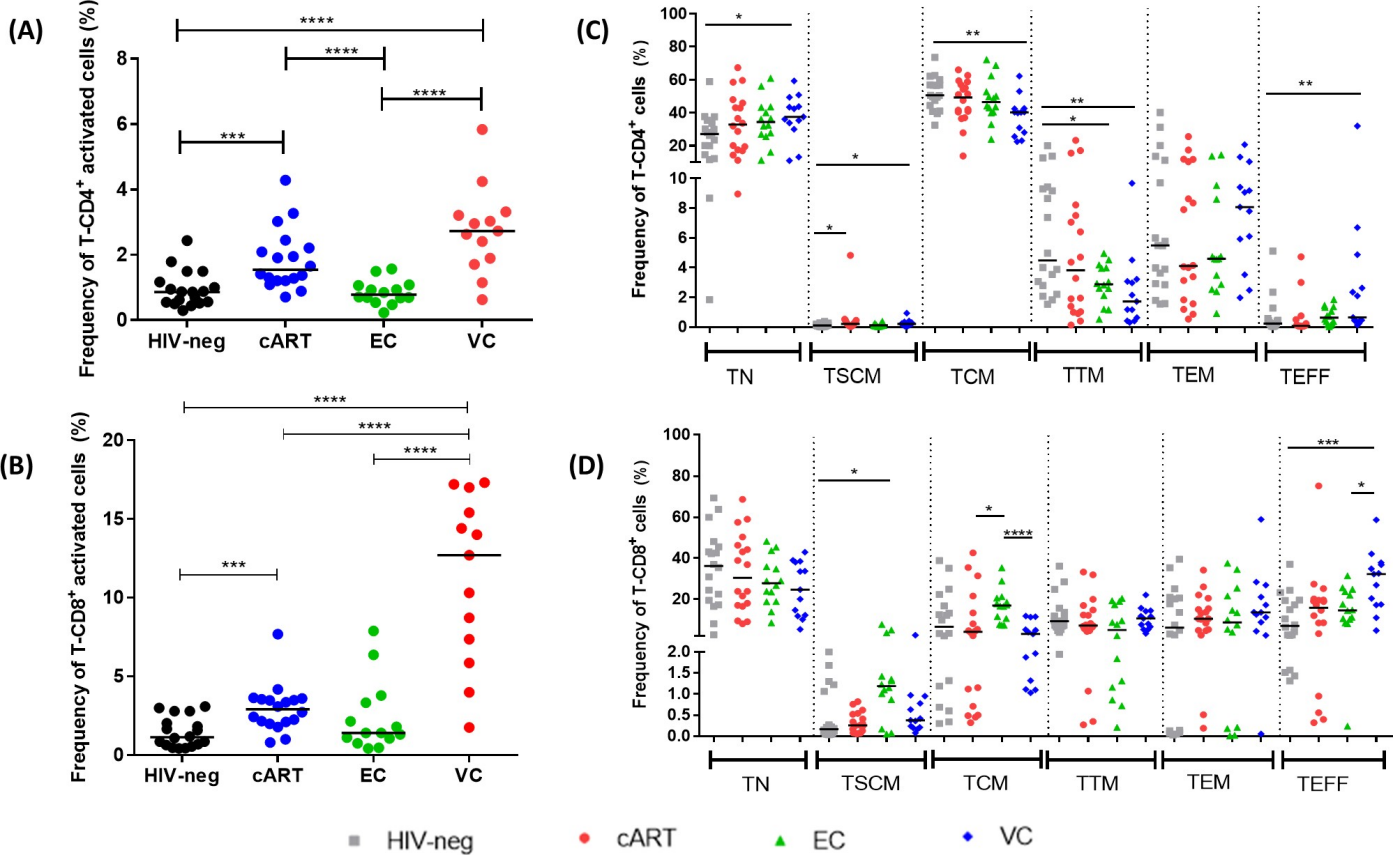
T cell activation levels and subset profiles in HICs and control groups. (A) Frequencies of activated CD4^+^ (CD38^+^HLA-DR^+^) T cells. (B) Frequencies of activated CD8^+^ T cells. (C) Frequencies of naïve (TN; CD45RA^+^CCR7^+^CD27^+^CD95^-^), stem memory (TSCM; CD45RA^+^CCR7^+^CD27^+^CD95^+^), central memory (TCM; CD45RA^-^CCR7^+^CD27^+^), transitional memory (TTM; CD45RA^-^CCR7^-^CD27^+^), effector memory (TEM; CD45RA^-^CCR7^-^CD27^-^), and effector (TEFF; CD45RA^+^CCR7^-^CD27^-^) CD4^+^ T cells. (D) Frequencies of TN, TSCM, TCM, TTM, TEM and TEFF CD8^+^ T cells. For panels C and D, gray squares represent HIV-neg, red circles represent cART, green triangles represent ECs and blue diamonds represent VCs; the horizontal line represents the median for the group; P-values were calculated using the Mann-Whitney test in GraphPad Prism and are represented as follows: * p < 0.05; **p < 0.01; ***p < 0.001; ****p < 0.0001.

When evaluating the level of activation in CD4^+^ T cells (CD4^+^CD38^+^HLA-DR^+^), ECs showed similar frequencies of activated cells as those observed for the HIV-neg group. On the other hand, the VC and cART groups presented a higher frequency of these activated cells when compared to the EC (p < 0.0001 for both groups) and HIV-neg (p < 0.0001 and p = 0.0003, respectively) groups (Fig 1A). In relation to activated CD8^+^ T cells (CD8^+^CD38^+^HLA-DR^+^), VCs presented higher levels of activation in comparison with all other groups (p < 0.0001 for ECs and HIV-neg; p = 0.0002 for cART) (Fig 1B). The cART group presented higher frequencies of activated CD8+ T cells only when compared with HIV-neg individuals (p = 0.0003). As observed for the activated CD4^+^ T cell subset, similar frequencies of activated CD8^+^ T cells were found in the EC and HIV-neg groups.

### Frequency of naïve, memory and effector CD4^+^ and CD8^+^ T cell subsets

Phenotypic analyses were performed to compare the frequencies of distinct T cell subsets (TN, TSCM, TCM, TTM, TEM, and TEFF) among the studied groups for both the CD4^+^ and CD8^+^ T cell compartments. When we evaluated CD4^+^ T cell subsets (Fig 1C), ECs presented similar levels of all subsets compared to HIV-neg, except for TTM cells, for which a significantly lower level was observed (p = 0.0304). VCs presented higher frequencies of TEFF (p = 0.0062), TN (p = 0.0111) and TSCM cells (p = 0.0315), but lower frequencies of TTM (p = 0.0032) and TCM cells (p = 0.0020) when compared to the HIV-neg group.

Among the CD8^+^ T cell subsets (Fig 1D), ECs presented higher frequencies of long-lived TSCM cells (p = 0.0139) than HIV-neg, while VCs presented a higher frequency of TEFF cells (p = 0.0007) in comparison to HIV-neg.

### Frequency of total Treg cells and Treg subsets

**Fig 2:**
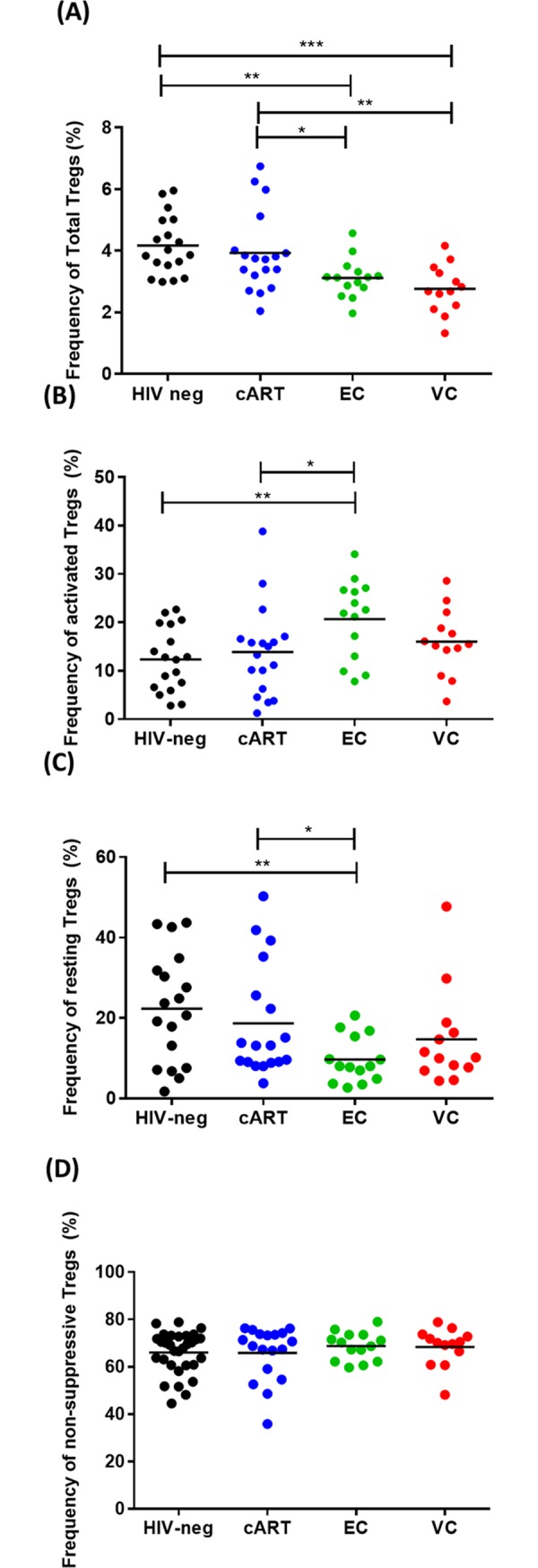
Treg cells subset profiles in HICs and control groups. (A) Frequencies of total Treg cells (CD4+CD25highFoxp3+). (B) Frequencies of activated (CD45RA^-^Foxp3^high^) Treg cells. (C) Frequencies of resting (CD45RA^+^Foxp3^low^) Treg cells; (D) Frequencies of non-suppressive (CD45RA^-^Foxp3^low^) Treg cells. The frequencies of activated, resting and non-suppressive Treg cells are relative to those of total Treg cells. P-values were calculated using the Mann-Whitney test in GraphPad Prism and are represented as follows: * p < 0.05; **p < 0.01; ***p < 0.001; ****p < 0.0001.

We evaluated the frequency of total Treg cells, and their activated, resting, and non-suppressive subsets (Fig 2). Both ECs and VCs presented lower levels of total Treg cells compared to HIV-neg (p = 0.0018 and p = 0.0001, respectively) and cART groups (p = 0.032 and p = 0.006, respectively) (Fig 2A). When analyzing the Treg subsets, ECs presented higher frequencies of activated Treg cells than the cART (p = 0.037) or HIV-neg (p = 0.003) groups (Fig 2B) and, inversely, a lower frequency of resting Treg cells than the cART (p = 0.008) or HIV-neg (p = 0.036) groups (Fig 2C). Similar frequencies of non-suppressive Treg cells were observed for all groups (Fig 2D). We observed an inverse correlation between total Treg and CD8^+^ T cell activation (r = -0.3607; p < 0.004), but we did not detect correlations between Treg subsets and CD4^+^ T cell activation (S1 Fig).

### Frequency of Th17 cells and Th17/Treg ratio

**Fig 3:**
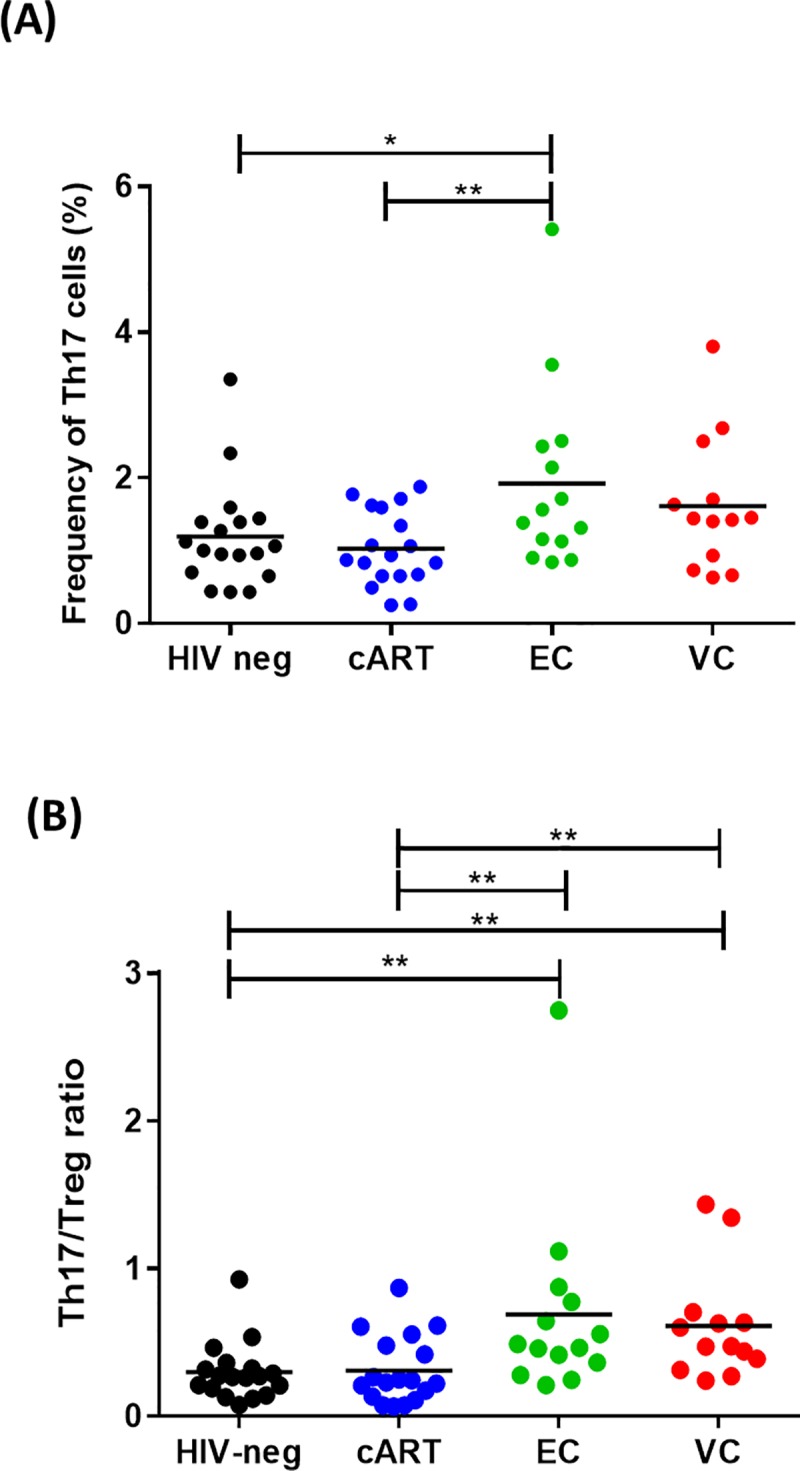
Th17 cell frequencies and Th17/Treg ratio in HICs and control groups. Frequencies of Th17 cells (CD4^+^IL17^+^) among the studied groups are shown in the graph. (B) Th17/Treg ratios are shown in the graph. The Th17/Treg ratio was calculated by using the frequencies of both populations concerning the CD4+ compartment. P-values were calculated using the Mann-Whitney test in GraphPad Prism and are represented as follows: * p < 0.05; **p < 0.01; ***p < 0.001; ****p < 0.0001.

We also analyzed the frequencies of Th17 cells and the Th17/Treg ratio among the studied groups (Fig 3). ECs presented higher levels of Th17 cells and Th17/Treg ratios when compared to the HIV-neg (p = 0.048 and p = 0.002) and cART (p = 0.009 and p = 0.007) groups. Although no statistically significant differences were observed when VCs were compared to the other groups, VCs presented higher Th17/Treg ratios than the cART (p = 0.004) and HIV-neg groups (p = 0.001). We observed an inverse correlation between Th17 frequencies and total Tregs (r = -0.2515; p = 0.04, S1 Fig).

## Discussion

In the present study, we evaluated parameters related to the immune activation state of T cells and the balance of Th17/Treg cells in HICs with different levels of viral replication control to evaluate immunologic factors related to the better infection control. Although most HIV-infected individuals present an immunological dysregulation characterized by alterations in the frequency of T cell subsets, excessive and systemic immune activation/inflammation and changes in the intestinal mucosa [2,4,60], HICs have a more preserved immunological system and represent a model of spontaneous infection control [61–63].

By evaluating the frequency of CD38^+^HLA-DR^+^ cells in both CD4^+^ and T CD8^+^ T cells, we identified higher levels of activated T cells in VCs compared to the other studied groups, indicating the contribution of viral replication to the increase in immune activation even among individuals with low but detectable viremia. Immune activation during chronic HIV infection is one of the major issues associated with viral persistence and disease progression, leading to CD4 T cell depletion, enhancement of viral replication, and exhaustion and senescence of T cells [2]. This setting results in an impairment of the immune response, despite the increased activity, as shown by the use of serum and cellular activation markers as predictors of AIDS [64–68]. We also observed higher frequencies of CD4^+^ and CD8^+^ TEFF cells in VCs, indicating an increase in T cell differentiation. These data highlight the need for increased care and surveillance of individuals with low-level viremia since even lower levels of antigenic stimulation have a negative effect on the immunological system.

Activation levels were lower among cART-treated patients than VCs, pointing to the undeniable positive effect of cART. In the last decades, drugs with higher genetic barriers and new regimens have been developed [69], bypassing drug resistance issues, improving the survival and quality of life of infected individuals [70–72] and decreasing transmission rates [73,74], which supports the expansion of cART coverage and early initiation. In this context, antiretroviral therapy could improve immunological health in viremic controllers, lowering activation levels as observed in cART individuals. On the other hand, activation levels in cART individuals were higher than those observed in HIV-negative individuals, consistent with previous studies that indicate that cART alone cannot normalize T cell activation [5–8,75]. These data reinforce the idea that, although driven by HIV infection, immune activation is boosted by factors that go beyond the direct effects of viral replication. Bystander activation of CD8^+^ T cells in HIV infection has been observed to be associated with the reactivation of other viruses [76,77] and with the circulation of proinflammatory cytokines [78,79], while microbial translocation due to CD4^+^ T cell depletion in the gut mucosa is considered one of the major mechanisms driving immune activation [80–82]. Besides, suboptimal penetration of drugs in anatomical sites such as the central nervous system, GALT, and lymph nodes is associated with persistence of viral replication in those tissues despite plasma viral load <LDL [83–86].

Moreover, our study showed that ECs had low levels of activated T cells, similar to those observed for HIV-negative individuals. Although these results contrast with other studies that showed higher T cells activation in ECs [14–16], the normalized frequencies observed here in patients with long-term control of infection are a signal of immune preservation at a magnitude that is not achieved even with antiretroviral therapy, as most of our studied individuals had long-term HIV infection. The higher frequencies of CD8^+^ TCM cells in ECs found in our study also point towards this hypothesis, as others have shown the importance of this population to the maintenance of the immune response [87,88]. Also, lower activation levels may not impair the immune response against HIV as other studies have shown that, despite the activation levels, ECs present efficient cytotoxic and HIV-specific response [89]. Together, these data suggest a better immune response in ECs related more to increased efficiency than to increased magnitude.

In addition to the increase in CD8^+^ TCM frequency when compared with that in cART, we also detected an increased frequency of TSCM cells in ECs compared with HIV-neg. TSCM cells were identified as memory T cells characterized by the increased expression of naïve markers and presenting an increased proliferative capacity and self-renewal potential [90]. Despite the susceptibility of TSCM cells to HIV-1 infection [91], the proportion of CD8^+^ TSCM cells has been previously inversely correlated to viral replication, and immune activation [92], which is in agreement with our study, and the preservation of the CD4^+^ TSCM population was associated with a better prognosis in both HIV-1 and SIV infection [92,93]. Our data here also support the association between the maintenance of CD4^+^ TSCM cells at normal levels with better control of infection.

In addition to alterations in classical naïve, memory and effector T cell subsets, we also investigated the frequencies of both Th17 and Treg cells, as these cells influence the activation of effector T cell profiles in different settings. In our study, we observed lower frequencies of total Treg cells in both HIC groups when compared to the cART and the HIV-neg groups. We also observed a negative correlation between the frequency of total Tregs and activated CD8^+^ T cells, highlighting the immunosuppressive function of these cells. While this association indicates a positive effect for the increase in the frequency of these cells to control the exacerbated immune activation due to HIV infection [28,35,42], several studies have shown a correlation between higher Treg frequencies and increased viral load and progression to AIDS [19–28].

Although this duality indicates negative effects in the long term in the context of HIV infection, the relationship between Tregs and immune activation could be a useful tool for the development of alternative strategies aiming at reservoir elimination [94]. The depletion of Tregs could be used as a latency reversal strategy to induce HIV replication from reservoirs, contributing to the “shock” needed in “shock and kill” strategies. For example, Treg depletion in HIV-infected humanized mice led to viremia rebound under cART followed by a reservoir decrease in lymphoid tissue [95], while Treg depletion in the NHP model lead to viral rebound and increase in the SIV-specific response [96].

Although some studies observed higher Treg frequencies or absolute counts in HICs compared to HIV-negative individuals [32,97], the majority showed lower [98,99] or similar [33,34,42,89,100] levels of Treg cells among HICs vs. healthy subjects. In this context, the low frequencies of Treg cells in HICs observed in this study and others point towards the preservation of immune responsiveness in these individuals.

Based on CD45RA and Foxp3 expression, Treg cells can be further separated into three different subsets: activated, resting, and non-suppressive Treg cells. Despite the lower frequencies of total Tregs, we observed an increased frequency of activated Tregs and a decrease in resting Tregs in ECs when compared to control groups, as it was observed by Gaardbo et al. [101]. Together, these results indicate that the balance between the different Treg subsets could have an important role in HIV pathogenesis and that the influence of Tregs on disease progression goes beyond the increase in the total Treg population.

Evidence of a preserved immune system in HICs was also observed for Th17 cells. Here, we observed higher frequencies of these cells in the peripheral blood in both ECs and VCs compared with both the HIV-neg and cART groups. Th17 cells are important in the context of HIV infection due to their participation in the host defense processes against several pathogens in the gut tissue. Besides, Th17 cells also induce epithelial regeneration [49], helping to maintain the physical integrity of the mucosal barrier. The GALT is a major site of HIV replication and suffers a massive depletion of CD4^+^ T cells early in the infection [102,103]. This setting leads to a pro-inflammatory state that disrupts the gut mucosal barrier and enhances microbial translocation. The increase in microbial translocation, as previously stated, is believed to be one of the most significant causes of the increased immune activation observed in HIV-infected patients [80,81,104], highlighting the importance of Th17 cells for the control of immune activation in the context of HIV infection.

In general, frequencies of Th17 cells correlate negatively with the plasma viral load and positively with CD4^+^ T cell counts, and low frequency of this subset has been observed in HIV-infected patients with progressive disease [33,53–56,105], indicating impairment of the gut immune response. Falivene et al. demonstrated the prognostic value of Th17 cell frequency, showing that lower frequencies of Th17 cells and higher frequencies of activated cells were observed in acutely infected individuals who progress faster to AIDS [33]. In contrast, higher baseline Th17 frequencies in individuals undergoing acute infection are associated with enhancement of the HIV-specific T cell response [33]. Among HICs or LTNPs, frequencies of Th17 cells are normally similar to those observed in HIV-1-uninfected individuals [33,34,54,106]. In the present study ECs, but not VCs, showed higher frequencies of Th17 cells compared to the HIV-neg and cART-treated controls, indicating a protective role of Th17 cells in HIV-1 infection.

Beyond the individual dynamic of Th17 and Treg subsets, we also observed an inverse correlation between the frequency of Th17 and Treg cells, as expected, since these cells share development pathways [57]. Our data regarding the Th17/Treg ratio on both HIC groups agreed to previous observations that found higher ratios in individuals with natural control of infection in comparison to typical progressors or HIV-negative individuals [32–34]. Overall, this preservation of the Th17/Treg ratio in both HIC groups indicates the preservation of the immune response.

## Conclusions

Our data showed that ECs have low levels of activated T cells and a high frequency of activated Treg cells, which can contribute to lower immune activation in these individuals. In addition, the high frequency of Th17 cells in ECs can be indicative of a preserved mucosal response.

## Supporting information

S1 Fig Significant correlations involving the frequency of total Tregs. **(**A) Correlation between the frequencies of total Tregs and activated CD8+ T cells. (B) Correlation between the frequencies of total Tregs and Th17 cells. R and p-values are shown for each correlation. Dots related to each studied group are coloured according to legend on Fig 1.
(TIF)

S1 Table Raw data obtained at the study. (XLSX)
