# Supplementary figures and images for: HIV-1 elite controllers present a high frequency of activated regulatory T and Th17 cells
Source: PLoS One. 2020 Feb 5;15(2):e0228745. doi: 10.1371/journal.pone.0228745 (PMC7001932; doi:10.1371/journal.pone.0228745)

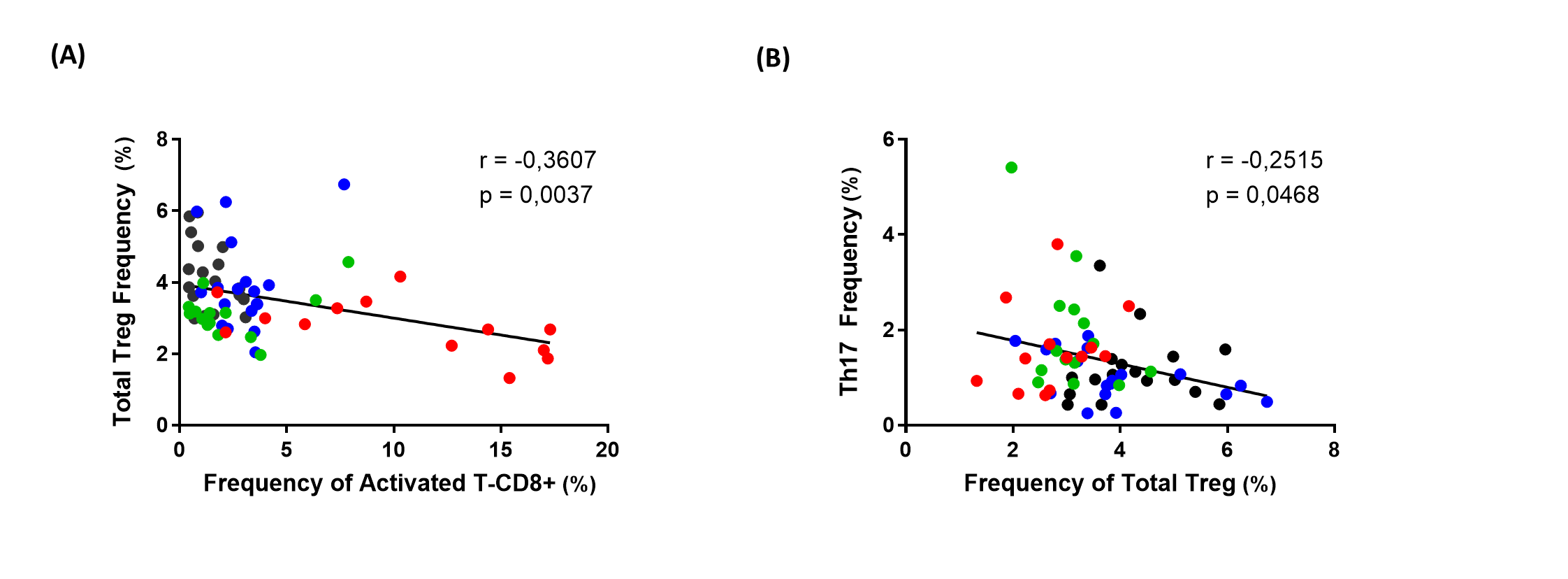

Supplement: S1 Fig — (A) Correlation between the frequencies of total Tregs and activated CD8+ T cells. (B) Correlation between the frequencies of total Tregs and Th17 cells. R and p-values are shown for each correlation. Dots related to each studied group are coloured according to legend on Fig 1. (TIF) [file pone.0228745.s001.TIF]
